# Evaluation of an occipito-cervico fusion with a new implant design: a biomechanical study

**DOI:** 10.1186/s12891-021-04112-z

**Published:** 2021-03-06

**Authors:** Filippo Migliorini, Alice Baroncini, Yasser El Mansy, Valentin Quack, Andreas Prescher, Max Mischer, Johannes Greven, Markus Tingart, Jörg Eschweiler

**Affiliations:** 1 Department of Orthopaedic Surgery, RWTH Aachen University Hospital, Pauwelsstraße 30, 52074 Aachen, Germany; 2 Department of Trauma Surgery, RWTH Aachen University Hospital, Aachen, Germany; 3 Institute of Molecular and Cellular Anatomy, RWTH Aachen University, Aachen, Germany

## Abstract

**Background:**

A novel implant for occipitocervical fusion consisting of a median plate with an additional hook inserting in the foramen magnum was tested. Aim of this study was to test the stability of a new implant for occipitocervical fusion against the already available and employed median plate implant without hook.

**Material and method:**

36 rigid polyurethane foams occipital artificial bones were used. The two occipital implants, namely the occipital plate with hook (Group 1) and the one without hook (Group 2), were applied to the artificial occiput trough three occipital screws and ensured into the experimental setup trough a crossbar. The test parameters were set using the testing machine software as follows: (1) test speed: 10 mm/ min, with 25 mm/ min maximum; (2) preload: 5 N; (3) force switch-off threshold: 90% force drop from F_max. Failure force and path were recorded. Failure force is defined as the maximum reaction force under which failure occurs (F_max), while failure path is the travel path during which failure occurs (dL).

**Results:**

Group 1 (plate with hook) showed a mean failure force of 459.3 ± 35.9 N and a mean failure path of 5.8 ± 0.3 mm Group 2 (plate without hook) showed a mean failure force of 323.9 ± 20.2 N and a mean failure path of 7.2 ± 0.4 mm. The Shapiro-Wilk test score was not significant (*P* >  0.1), assuming that data were normally distributed. Group 1 had a statistically significant greater F_max (+ 135.37; *P* >  0.0001) and less dL (− 1.52; *P* > 0.0001) compared to group 2.

**Conclusions:**

Medial plates with foramen magnum hooks showed to be more stable that plates without a hook. These new implants may represent a new tool in OCJ fixation, but further studies are required to investigate their behavior in an anatomical setting.

## Background

The occipitocervical junction (OCJ) is the transitional region between the cranium and the spine and represents the most mobile area of the cervical spine. The occiput (C0)-C1 joint is the biggest contributor to the flexion and extension movements (21° and 3.5°, respectively), while the segment C1-C2 is responsible for axial rotation (about 25–35° per side) [1]. This complex anatomical region is rich in membranes and synovial joints [1]. Posterior occipitocervial arthrodesis in indicated for in stability of the OCJ [2, 3]. Given the complex anatomy and biomechanics, the rate of failure for external fixators for OCJ instability is up to 30% [4] Thus, a novel implant for a more stable internal, surgical fixation is required. 

Concentrating on the occipital part of the implants, instrumentation of this area represents a challenge for the surgeon due to its anatomical peculiarities [4, 5, 6]. The occipital protuberance is its thickest bony part, but the thickness is not uniform and gradually decreases laterally and caudally [7], so that in some settings even the shortest provided screws may be too long or the length of the screw would not provide sufficient stability [8]. Many occipital implants have been proposed, but no consensus has yet been reached [4, 8]. Techniques that have been employed include the use of rods attached to wires and onlay bone grafting with wire fixation. Hook and rod constructs were attempts at mitigating wire-cutting complications while providing stability to the cervical spine, and OCJ, respectively [9]. Most screw/plate constructs allow a degree of rotation of the screw head in respect to the plate, which over time can lead to reduction of the implant stability and, eventually, pullout [4]. For this reason, and also to reduce iatrogenic complications such as lesion of the meningeal sheets or the venous plexus alternative implants such as occipital-condyle screw plates or bone hooks have been developed [8, 10, 11].

In this work, we present a novel implant (Medicon, occiput plate) consisting of a median plate with a hook inserting in the foramen magnum. Historically, stabilization of the craniocervical junction consisted of onlay bone grafting with posterior wiring. Aim of this study was to test the stability of a new implant with an additional hook for bone fixation against the already available and employed median plate implants.

## Methods

### Implants and test specimen

**Fig. 1:**
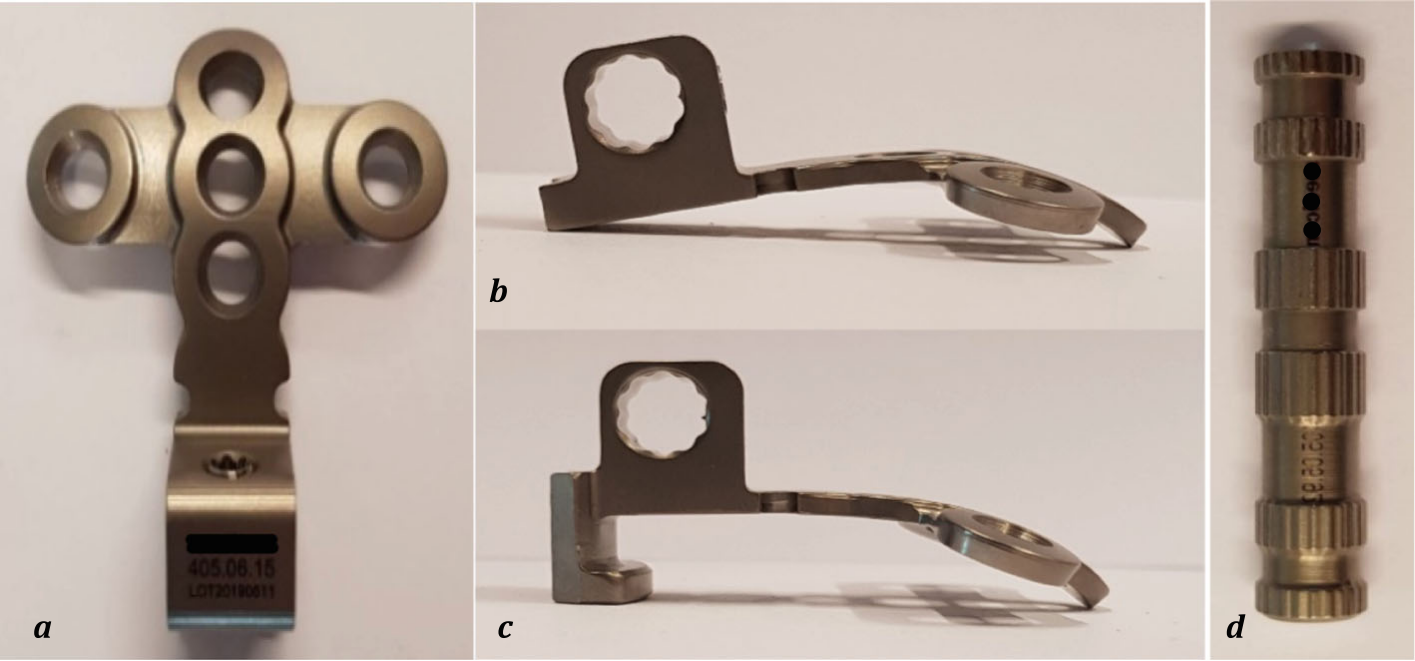
Occipital plate (**a**: dorsal view; **b**: lateral view without hook; **c**: lateral view with hook; **d**: crossbar)

For implant testing, 36 artificial occipital bones made of rigid polyurethane foams (Sawbone®, Pacific Research Laboratories Inc., Vashon, Washington, USA) that complied with the requirements ASTM F1839–08 [12] were used. Eighteen artificial bones were available for each of the two examined group. The two occipital implants (Fig. 1) (Medicon, occiput plate, made of Ti-6AI-4 V surgical alloy), namely the occipital plate with hook (Group 1) and the one without hook (Group 2), were applied to the artificial occiput trough three occipital screws. The implants were manufactured via milling without a special postprocessing. The implants were fixed by an experienced surgeon. Other than the presence of the hook, the plates were identical in shape and material. The construct consisting of artificial occiput, plate, screws and crossbar were ensured into the experimental setup trough a crossbar (Fig. 1).

### Experimental test setup

**Fig. 2:**
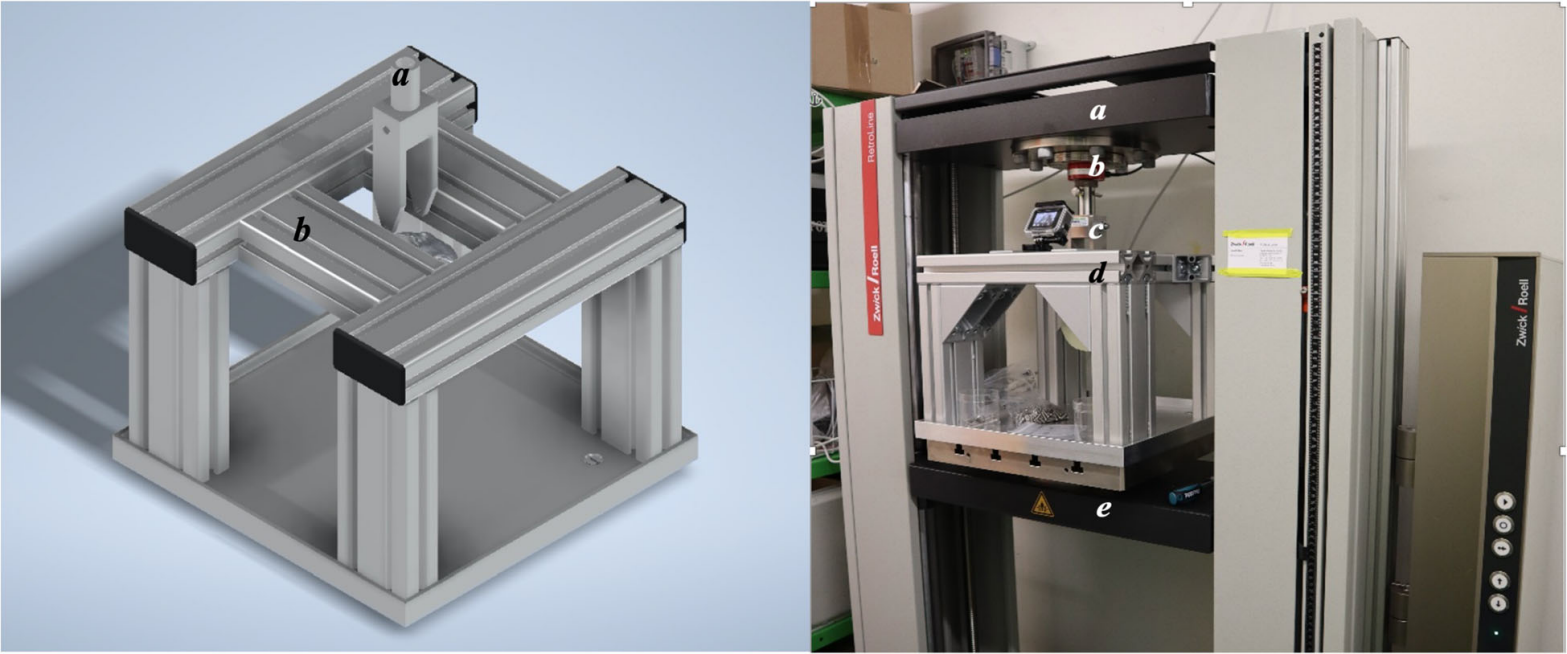
Left: CAD model of the experimental setup (**a**: fastening claw; **b**: frame construction); right: test set-up (**a**: upper traverse; **b**: Force transducers; **c**: frame construction; **d**: under traverse)

An experimental test setup was developed to examine and analyse the implants (Fig. 2). The experimental setup consisted of aluminum profiles (Item - Solingen, Germany), which were connected together to function as frame to fix the artificial bone. A fastening claw made of two force carriers was used to clamp the crossbar and served as a connecting piece between the force transducer and the implant. Axial pullout forces were applied to the implants on all artificial bones using a custom-made jig attached to a servohydraulic universal material testing machine (MTM) (Zwick and Roell - Ulm, Germany). The force transducer of the MTM was used to measure the applied force: with an accuracy class of 1, this instrument has a maximum measurement deviation of 1%.

### Preparation

**Fig. 3:**
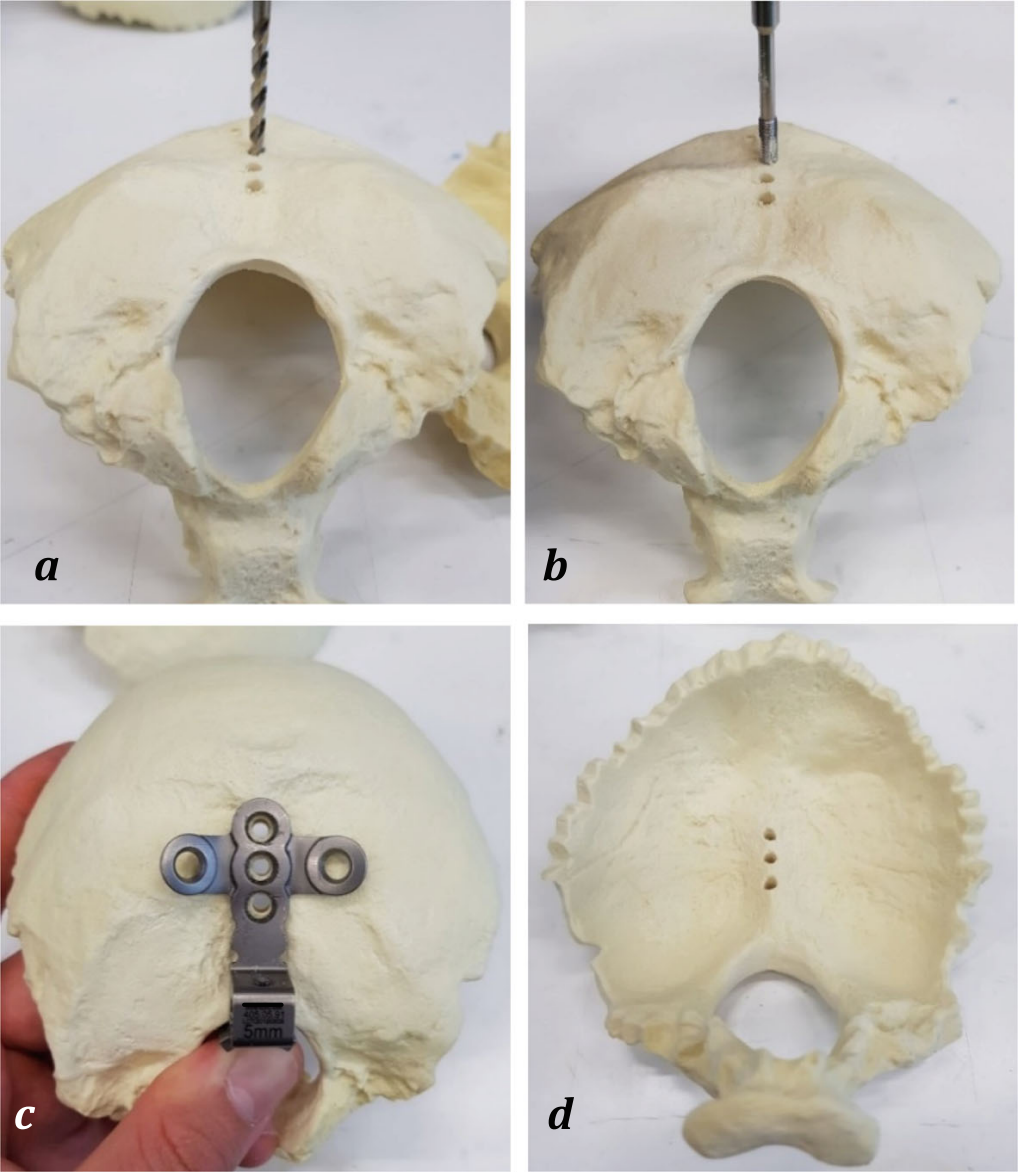
With a drill, the diameter of which is 3.5 mm, three through-holes are made in the occiput orthogonally to the top of the plate (**a**); each drill hole is then provided with a thread with the thread cutter (**b**); the bores are concentric to the bores of the implant (**c**); artificial occiput with through-holes along the internal occipital crest (**d**)

The same preparation procedure was used for both experimental groups. First, a template implant was placed above the artificial occiput to mark three central drill holes along the internal occipital crest. Then an awl was pierced into the three markings to ensure precise placement of the holes. With a 3.5 mm drill, three holes were made in the artificial occiput orthogonally to the long axis of the plate (Fig. 3a). Each drill hole was then provided with a thread with the thread cutter (Fig. 3b). The implants of each test group were then positioned on the artificial occiput so that the three holes were centered concentrically one above the other (Fig. 3c, d). Then three occipital screws were screwed in with a hand screwdriver and tightened with a torque of 1 Nm using a torque screwdriver (Wera - Wuppertal, Germany). Finally, the crossbar was pushed laterally into the plate and fixed with a torque of 1.6 Nm using a torque limiter (W + S Solutions - Tuttlingen, Germany).

### Testing

**Fig. 4:**
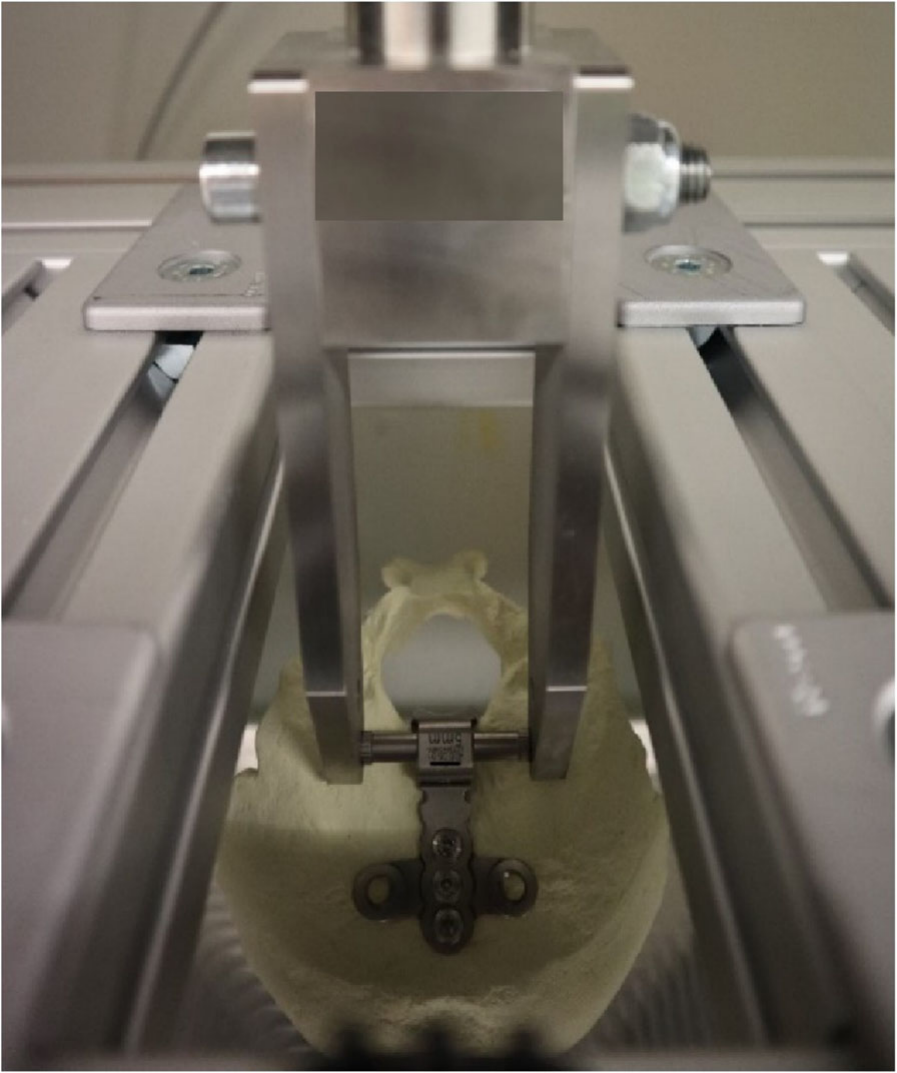
The crossbar is pushed into the through holes of the two force carriers of the fastening claw

The implant construct consisting of the artificial occiput, plate, screws and crossbar was ensured into the experimental setup. The crossbar was pushed into the holes of the two force carriers of the fastening claw (Fig. 4). The artificial occiput was then placed in the center of the experimental setup and the crossbar was fixed to prevent it from slipping sideways. Afterwards, the test parameters were set using the testing machine software as follows: (1) test speed: 10 mm/ min, with 25 mm/ min maximum (according to ASTM 2706 [13]); (2) preload: 5 N; (3) force switch-off threshold: 90% force drop from F_max.

A GoPro camera (San Mateo, California, USA) was installed to document every measurement. 36 measurements were made (18 tests for each group). Failure force and path were recorded with the MTM. Failure force is defined as the maximum reaction force under which failure occurs (F_max), while failure path is the travel path during which failure occurs (dL). The information from the GoPro camera were just taken to document the process of pull out testing and to ensure the safety of the examiner. Furthermore, the information from the GoPro camera should be used to follow the pull out process in case of questionable results to have a chance to recapitulate the process afterwards. With this intention, no synchronization was needed between MTM and GoPro.

### Statistical analysis

The statistical analysis was performed using the IBM SPSS software version 25. Mean, standard deviation and difference for F_max and dL were evaluated. The standard error (SE) was evaluated. The confidence interval (CI) was set at 95%. The Shapiro-Wilk test was used to check if variables F_max and dL had normal distributions, with values of *P* > 0.1 considered satisfactory. The unpaired t-test was used to determine whether there was a significant difference between the two test groups, with *P* < 0.05 considered statistically significant.

## Results

**Fig. 5:**
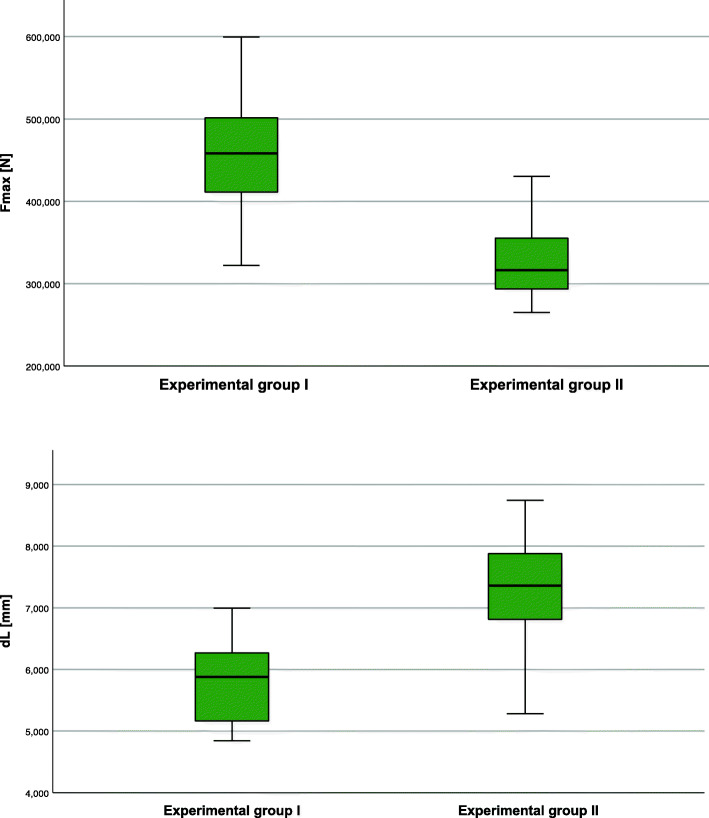
Comparison of the failure forces and path of both test groups

**Fig. 6:**
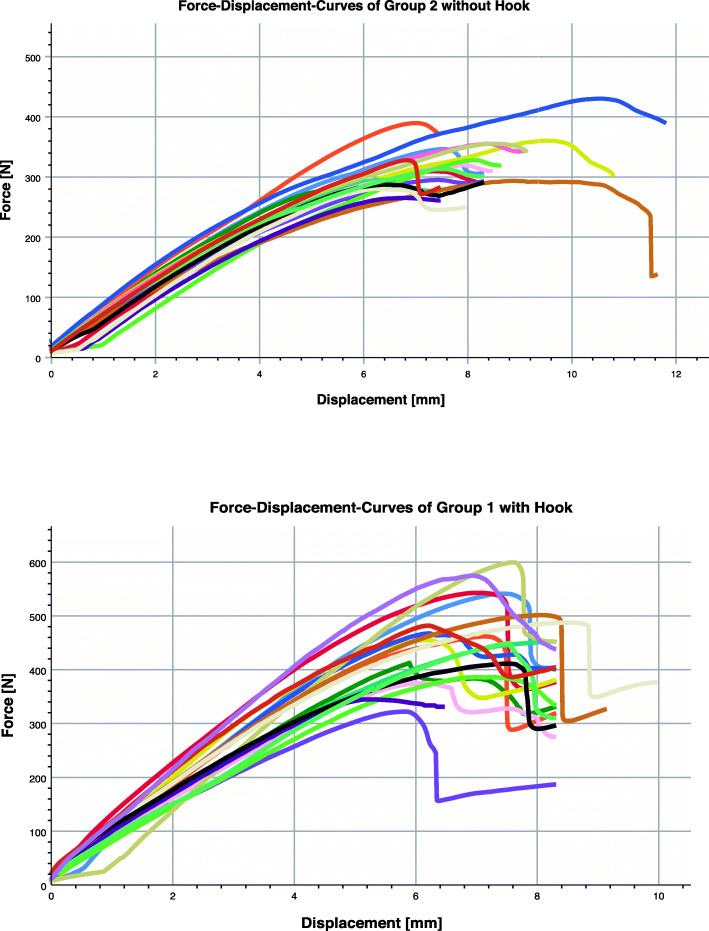
Force - Displacement diagrams with and without hook (each test is reported in a different colour)

Group 1 (plate with hook) showed a mean failure force of 459.3 ± 35.9 N and a mean failure path of 5.8 ± 0.3 mm. Group 2 (plate without hook) showed a mean failure force of 323.9 ± 20.2 N and a mean failure path of 7.2 ± 0.4 mm (Table 1). The Shapiro-Wilk test score was not significant (*P >* 0.1), assuming that data were normally distributed. Group 1 had a statistically significant greater F_max (+ 135.37; *P* > 0.0001) and less dL (− 1.52; *P >* 0.0001) compared to group 2. Box plots of failure forces and paths are shown in Fig. 5, while Fig. 6 shows the different force-displacement curves. Data from this study demonstrate that the hook had a better performance concerning applied force.

**Table 1:** Overall results

Variable	Test group I (***n = 18***)	Test group II (***n = 18***)	Difference	SE	95% CI	***P***
F_max	459.3 ± 76.2	323.88 ± 42.9	135.37	20.616	93.47 to 177.27	> 0.0001
dL	5.82 ± 0.7	7.24 ± 0.8	−1.42	0.256	−1.94 to − 0.89	> 0.0001

## Discussion

**Fig. 7:**
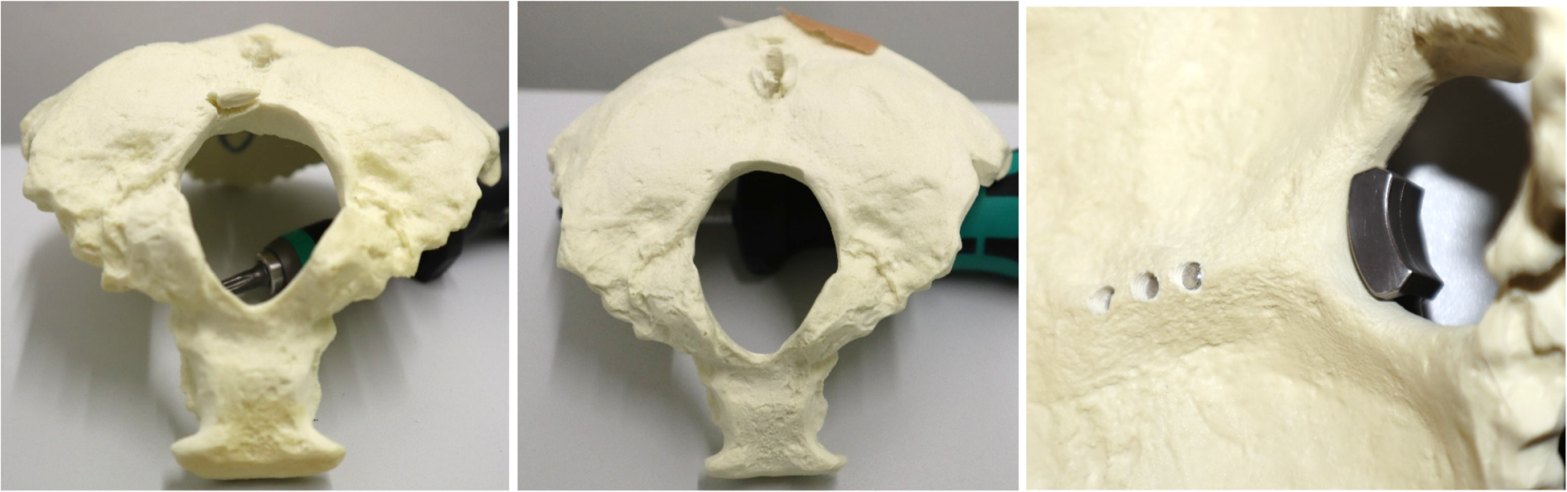
From left to right: Broken edge of the occipital squama due to the hook breaking off; Deformed edge of the occipital squama due to the hook slipping; The shape of the hook is not adapted to the bone structure

The occiput bone is flat and protects the contents of the posterior fossa, and furthermore, encircles on three sides the foramen magnum [4]. The occipitocervical junction is characterized by an extremely mobile transition segment of the upper spine and the interface between head and spine, respectively [9]. The function of the occipitocervical junction can impose significant stress under abnormal motion, trauma, or various spinal pathologies at that level [9]. Indications for occipitocervical junction fusion include traumatic injuries, tumors, degenerative instability, rheumatoid disease, and congenital anomalies of the occipitocervical junction. If surgical intervention is necessary, stabilization of the occipitocervical junction requires a robust fixation with an adequate implant. Many currently available fixation implants present an integrated plate-rod system, that often present difficulties for insertion of the C1 and C2 screws and require extensive bending to be adapted to the occipital bone [14]. Furthermore, these systems require a paramedian fixation of the screws, that thus cannot be placed in the thickest, median area of the occipital bone [14]. Newer, non-integrated implants present an attachment system between the occipital plate and the cervical rods and allow independent fixation of the cervical and occipital screws; however, screw placement around the midline limits the number of the screws that can be implanted [7]. For a safe and stable fixation we introduced a new, non-integrated implant with an additional hook for higher impact: the hook provides stability despite the use of a reduced number of occipital screws, while the non-integrated construct allows for implant feasibility. We found out that the additional hook in the occipital implant lead to a significantly higher failure force, and, at the same time, to a significantly lower failure path. As shown in Fig. 5, the mean failure force of test group 1, at 459.3 N, is above the mean failure force of test group 2. Using the t-test, a significant difference between the mean values of both groups was demonstrated. The reason for the higher failure force is most likely the additional hook of the plate implant. The additional hook around the edge of the Squama occipitalis offers an additional form fit between the implant and the bony structure, that sums up with the frictional connection due to the screw, and ultimately leads to a higher pull-out or failure force. Furthermore, shown in Fig. 5, the measurement data from test group 1 had a greater spread than that of test group 2 (323.9 N). A possible explanation for this is provided by the different types of failure observed during the experiments. Most of the measurements resulted in the loss of the form fit due to the hook slipping out of the foramen magnum. In the remaining measurements, however, the hook broke out on one side at the edge of the squama occipitalis (Fig. 7). The different behavior of the implants during the test can possibly be explained by a poor anatomical adaptation of the hook to the edge of the squama occipitalis (Fig. 7).

In summary, it can be said that the additional hook of the implant brings an increase in the failure force due to the additional form fit between the implant and the bone. However, due to the shape of the hook, there is no reliable form fit, so that premature failure of the connection cannot be ruled out if the hook slips out. If the hook slips prematurely, the implant only shows the performance of an implant without a hook.

A comparison of the failure paths also shows that the mean failure path of test group 2 is 7.2 mm, significantly higher than the mean failure path of test group 1 (5.8 mm). A possible explanation for this finding is also provided by the operating principle of the connection between the implant and the artificial bone. Due to the lack of form fit in test group 2, the implant tends to shift even under low loads. As a result, by the time the connection fails, a greater distance has already been traveled.

**Fig. 8:**
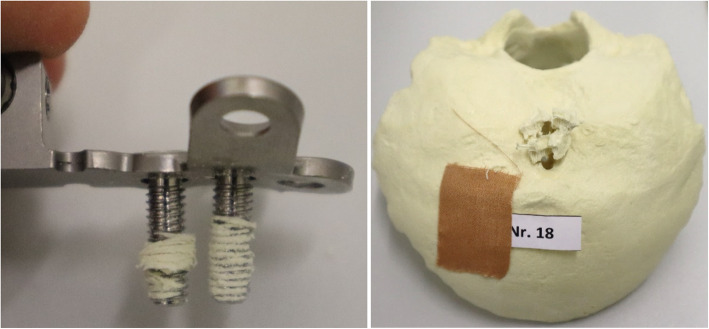
Threads filled with artificial bone as a result of shearing (left); Fragments of the artificial bone as a result of the screws breaking out (right)

As shown in Fig. 6, the spread of test group 2 is greater than that of test group 1. One possible explanation for this is the fact that the plate implant is connected to the occiput via three screw connections. Due to the lack of hooks on the implants of test group 2, the screws are subjected to tensile stress from the start of the test. Depending on the nature of the polyurethane foam, the tensile load causes the threads cut in the artificial bone to shear off and various screws to break out of the artificial bone. A failure therefore occurs under different failure paths (Fig. 8).

Beyond the biomechanical aspect, the use of a foramen magnum hook allows the use of an anatomical structure to additionaly anchor the construct, without requiring the creation of boreholes that may potentially lead to a damage to nervous or vascular structures. On the other end, cadaveric studies are required to investigate potential conflict of the hook with nervous structures in the foramen magnum, most of all in case of trauma or displacement.

Data presented here are from artificial bones (Sawbone®). The redeuced costs and time saved are some of the advantages of Sawbone®. Sawbones® offer the possibility to improve hands-on skills for residents surgeons, to aquire/implement the physical abilities on new surgical tasks for surgeons [15, 16]. Sawbones® demonstrated to be a reliable alternative to the fresh cadaveric speciements for biomechanical testing purposes, and have been widely employed in several experimental settings [16–21]. On the other hand, Sawbones® offer a uniform and consisten density [22]; thereby, it suppresses inter-specimen variability that occur in cadaveric experimental tests [23–25]. Thus, whist results may be more accurte, on the other hand they do not fully minic the heterogeneous caderic bone features. Furthermore, Sawbones® lack vasculonervous and soft tissues structures, along with the lack of accurate proprioceptive feedback. Further associated limitations do apply, including that realistic loosening associated with physiologic loading is not present. Also bony remodeling does not take place at the implant interface. Another limitation of the hook implant is the lack of personalization. It is known that a mismatch between hook and bone thickness can limit the stability of the implant [8]. Due to the inadequate anatomical adaptation of the hook to the bone structure can slip off from the foramen magnum and lead to a loss of the observed biomechanical advantages. The redesign of the hook according to the anatomical conditions would lead to a more reliable form fit and thus to a more reliable fixation. Such a customized anatomical adaptation of the hook could potentially be a next step here – however, the time required to produce a customized implant may not be compatible with surgical timing in an emergency situation. Furthermore, an angle-stable connection between the occipital screw and the plate implant could be considered to optimize the implant. As the studies by Cronier et al. [26] show, the use of an angle-stable connection could further increase the failure force and thus achieve an overall more stable implant construct. Furthermore, complications arose from the inadvertent compression of implants on the cervical spinal canal [9]. It was discovered that these additional hook for the cervical spine may present complications not only because of proximity to the spinal cord but also because of the necessity of the hooks to firmly seat on the lamina [9]. Given these limitations, results form the present study must be interpret with caution. However, results from the present study should encourage future studies to investigate the behavior of this innovative implant for occipitocervical fusion in a cadaeric human setting, overcoming limitations of the Sawbone® models.

## Conclusion

Medial plate with foramen magnum hook showed to be more stable that the plate without a hook. These new implants may represent a new tool in OCJ fixation, but further studies are required to investigate their behavior in an anatomical setting.

## Data Availability

The datasets generated during and/ or analysed during the current study are available from the corresponding author on reasonable request.
